# Depression, anxiety, and stress among adults in Kuwait across pre- and post-vaccine phases of the COVID-19 pandemic: a repeated cross-sectional study

**DOI:** 10.3389/fpubh.2026.1797542

**Published:** 2026-05-05

**Authors:** Eiman Alawadhi, Mohammad Almari, Marwan Al-Sharbati, Esra Al Khasawneh, Eleni Tolma

**Affiliations:** 1 Department of Epidemiology and Biostatistics, College of Public Health, Kuwait University, Kuwait City, Kuwait; 2 Department of Health Policy and Management, College of Public Health, Kuwait University, Kuwait City, Kuwait; 3 Department of Social and Behavioural Sciences, College of Public Health, Kuwait University, Kuwait City, Kuwait; 4 Department of Primary Care and Population Health, University of Nicosia Medical School, Engomi, Cyprus

**Keywords:** anxiety, coping strategies, COVID-19, depression, Kuwait, stress, vaccination

## Abstract

**Background:**

The COVID-19 pandemic has significantly affected mental health globally, with important implications for pandemic preparedness, yet psychological responses across pandemic phases in the Gulf region remain underexplored. This study assessed mental health outcomes among adults in Kuwait across pre- and post-vaccine phases of the COVID-19 pandemic to identify factors that can guide preparedness strategies.

**Methods:**

Two independent web-based surveys were conducted in June 2020 (pre-vaccine phase) and August 2021 (post-vaccine phase). In total, 1,923 adults completed validated questionnaires, including the Depression, Anxiety, and Stress Scale (DASS-21). Outcomes were compared across periods using descriptive analyses, and multivariate logistic regression identified predictors of moderate-to-severe mental health symptoms.

**Results:**

Overall, 40.9% of participants reported depression, 36.0% anxiety, and 25.0% stress. Participants surveyed during the post-vaccine phase had higher adjusted odds of moderate-to-severe anxiety (OR = 2.14, 95% CI: 1.61–2.85) and stress (OR = 2.31, 95% CI: 1.73–3.07) compared with those surveyed during the pre-vaccine phase. Females, young adults (18–29 years), and non-Kuwaitis had greater odds of adverse mental health outcomes, with non-Kuwaitis showing 50% higher odds of depression. Lower confidence in the healthcare system was also associated with greater psychological distress. In contrast, faith-based coping was associated with lower odds of stress (OR = 0.30, 95% CI: 0.20–0.45), and time management practices were linked to reduced anxiety (OR = 0.73, 95% CI: 0.55–0.96). Adherence to mask use and hand hygiene was lower during the post-vaccine phase.

**Conclusion:**

Substantial mental health challenges were observed in both pre- and post-vaccine phases of the pandemic, particularly among women, younger adults, and non-citizens. Protective coping strategies, especially faith-based and social support mechanisms, should thus be integrated into pandemic preparedness alongside biomedical responses.

## Introduction

Mental disorders constitute a substantial component of the disease burden in the Eastern Mediterranean Region. Data from the Global Burden of Disease study indicate that mental disorders accounted for 5.6% of total disability-adjusted life years in the region in 2013, with depressive and anxiety disorders contributing the largest share (1). In Kuwait, a study of primary health care attendees reported that 42.7% screened positive for at least one psychiatric disorder, including depression (22.9%) and anxiety (17.7%) (2). These findings indicate a notable burden of common mental disorders in the region.

The COVID-19 pandemic posed an unprecedented challenge not only to public health systems but also to population mental health worldwide. In Kuwait, early and decisive government responses were implemented to curb transmission, including school and mosque closures, suspension of air travel, national lockdowns, and partial curfews. These measures substantially altered daily life, requiring rapid adjustment to remote work, home-based schooling, and prolonged social isolation.

Although essential for infection control, these prolonged disruptions were associated with marked psychological consequences. Studies from multiple settings reported elevated levels of stress, anxiety, and depression during the early stages of the pandemic when uncertainty was high and vaccines were unavailable (3–5). Contributing factors included fear of infection, uncertainty about disease outcomes, bereavement, and prolonged social isolation. A global meta-analysis reported pooled prevalence estimates exceeding 30% for both anxiety and depression (6), while quarantine-related restrictions were also shown to have substantial psychological effects (7). Consistent with this evidence, the World Health Organization estimated a 25% global increase in anxiety and depression during the first year of the pandemic (8).

The introduction of COVID-19 vaccines in early 2021 represented a major turning point, offering protection and a pathway toward social reopening. However, vaccine rollout was accompanied by mixed public responses, including hesitancy driven by safety concerns, misinformation, and conflicting health messages (9–11). This phenomenon has been described as an “infodemic,” reflecting the parallel spread of misleading information during the pandemic (12).

As the pandemic has transitioned beyond its emergency phase, understanding patterns of psychological vulnerability and resilience remains highly relevant for informing mental health support strategies in future pandemics or similar disruptions. However, limited data from Kuwait have examined whether psychological distress persisted or changed following vaccine availability. In this study, we examined mental health outcomes among adults in Kuwait across two pandemic phases: the pre-vaccine and post-vaccine periods. By comparing mental health outcomes across two independent pandemic phases, this study aims to inform future preparedness strategies in which mental health is integrated alongside biomedical responses.

## Methods

We conducted two independent repeated cross-sectional web-based surveys at distinct time points in Kuwait: June 2020 (pre-vaccination) and August 2021 (post-vaccination), to assess mental health outcomes across different phases of the COVID-19 pandemic. The first survey (*n* = 1,111) was conducted over a one-month period in June 2020 during the partial reopening phase of Kuwait’s lockdown, when curfews and movement restrictions remained in place and vaccines were unavailable.

The second survey (*n* = 812) was conducted over a one-month period in August 2021, when vaccines were widely accessible and restrictions were eased for vaccinated individuals. During this time, Kuwait’s vaccination coverage reached a significant milestone of approximately 66–70% for at least one dose and 55–60% for full vaccination, as documented in the Our World in Data database (13). The two survey waves recruited independent participants using convenience-based electronic distribution. As such, comparisons between waves reflect population-level differences across pandemic phases rather than longitudinal changes within the same individuals.

Participants were recruited using convenience sampling with snowball dissemination through electronic invitations distributed via email, WhatsApp, and Instagram. Because the survey link was disseminated through open electronic channels, the number of individuals who received the invitation could not be determined; therefore, a response rate could not be calculated. Eligible participants were adults aged 18 years or older who were residing in Kuwait at the time of survey completion. Only fully completed questionnaires were included in the analysis; surveys that were initiated but not completed were excluded. To minimize duplicate responses, the survey platform restricted multiple submissions from the same device/IP address.

The questionnaire was developed in English and translated into Arabic using forward and back-translation procedures. Participants were able to complete the survey in either English or Arabic according to their preference. Face validity of both versions was assessed by public health researchers, and the final instrument was pretested among 15–20 individuals from the target population to ensure clarity, appropriate wording, and completion time.

The questionnaire was adapted from previously validated instruments used in pandemic-related research (5). It collected information on sociodemographic characteristics, physical symptoms, contact history with suspected or confirmed COVID-19 cases, COVID-19–related knowledge and concerns, precautionary behaviors, and mental health status.

Mental health outcomes were assessed using the 21-item Depression, Anxiety, and Stress Scale (DASS-21), a validated self-report measure widely used in population mental health research (14). The scale comprises three subscales assessing symptoms of depression, anxiety, and stress over the preceding week. Subscale scores were calculated according to DASS-21 scoring guidelines and multiplied by two to obtain final severity scores. For regression analyses, scores were dichotomized as normal/mild versus moderate-to-extremely severe using standard DASS-21 thresholds (14). Moderate-to-extremely severe categories were defined as ≥14 for depression, ≥10 for anxiety, and ≥19 for stress. Internal consistency of the DASS-21 subscales was assessed separately for each survey wave using Cronbach’s alpha. Reliability coefficients were good in both periods. In June 2020 (pre-vaccination), Cronbach’s *α* was 0.82 for depression, 0.82 for anxiety, and 0.86 for stress. In August 2021 (post-vaccination), corresponding values were 0.85, 0.83, and 0.86, respectively. The DASS-21 has demonstrated strong internal consistency and construct validity across diverse cultural settings (15). The Arabic version has previously demonstrated acceptable psychometric properties in Arab populations, supporting its use in the Kuwaiti context.

Key predictor variables included coping strategies, precautionary behaviors, information sources, and perceptions related to COVID-19. Coping strategies were assessed using binary (yes/no) items indicating engagement in specific behaviors (e.g., strengthening faith, positive thinking, relaxation, social support, exercise, hobbies, and time management). Precautionary behaviors were measured using seven items assessing frequency (always, sometimes, never) of protective practices such as mask use and hand hygiene. Perceptions related to COVID-19 (confidence in the healthcare system, perceived likelihood of infection, perceived likelihood of survival, concern about family infection, and satisfaction with health information) were originally measured on ordinal scales and dichotomized for regression analyses. Confidence and satisfaction variables were categorized as lower (not confident/slightly confident or dissatisfied/neutral) versus higher (confident/very confident or satisfied/very satisfied). Perceived likelihood and concern variables were dichotomized as likely/very likely versus unlikely/neutral. “Do not know” responses were treated as missing in analytic models.

Sample size calculations were based on estimating the prevalence of moderate-to-severe psychological distress, assuming a prevalence of 26.2% reported in an early COVID-19 study from China (5), and further supported by similar findings from Saudi Arabia (16). With a 95% confidence level and 5% precision, the minimum required sample size was 296 participants per survey wave. To account for non-response and non-probability sampling, the target sample was increased by 20%. Recruitment continued beyond this minimum to ensure sufficient events per variable for multivariable logistic regression analyses, with events-per-variable ratios exceeding commonly recommended thresholds for stability. The study was therefore powered for prevalence estimation rather than for detecting specific between-period differences or individual predictor effects.

Data from the two independent survey waves were analyzed separately and compared across time points. Descriptive statistics summarized participant characteristics, COVID-19–related behaviors, and mental health outcomes. Analyses were conducted using complete-case approaches, with observations containing missing values on variables included in a given model excluded from that analysis. Differences between the pre- and post-vaccination periods were assessed using chi-square tests.

Multivariate logistic regression models were used to identify independent predictors of moderate-to-severe depression, anxiety, and stress. Candidate variables were initially examined in bivariate analyses. Variables with *p* < 0.10 were considered for inclusion in multivariable models; however, key sociodemographic variables (sex, age group, nationality, and survey period) were retained in all models regardless of statistical significance, based on *a priori* conceptual relevance. Model fit was assessed using likelihood ratio tests and the Hosmer–Lemeshow goodness-of-fit test. Multicollinearity was evaluated using variance inflation factors. All models demonstrated adequate goodness-of-fit (Hosmer–Lemeshow *p* > 0.05), and no evidence of problematic multicollinearity was observed (all variance inflation factors < 2). All analyses were conducted using Stata version 16.0, with statistical significance set at *p* < 0.05.

### Ethics statement

Ethical approval was obtained from the Ministry of Health Standing Committee for Coordination of Health and Medical Research in Kuwait and the Kuwait University Health Sciences Centre Ethical Committee (Approval No. 2020/152). All participants provided informed consent prior to participation, and all responses were collected anonymously.

## Results

### Participant characteristics and behavioral changes

Of the 1,923 adults surveyed, the majority were females (54%), Kuwaiti (89%), and aged 30–44 years (45%) (Table 1). Compared to the pre-vaccination period, the post-vaccination group had a higher proportion of older adults (aged 45–59 years) and fewer young adults (aged 18–29) (*p* < 0.001). Several other sociodemographic characteristics, including age distribution, governorate, employment status, household size, and prior COVID-19 infection, differed significantly between the two survey waves, indicating compositional variation between the independent samples.

**Table 1:** Associations between participant characteristics during COVID-19 pre-vaccination period (June 2020) and post-vaccination period (August 2021), in Kuwait.

Variables	Total	Prevaccination	Postvaccination	*p*-value
	No (%)	No (%)	No (%)	
Total	1,923 (100.0)	1,111 (57.8)	812 (42.2)	
Gender				
Male	876 (45.6)	538 (48.4)	338 (41.6)	0.003**
Female	1,047 (54.5)	573 (51.6)	474 (58.4)	
Age (years)				
18–29	490 (25.48)	336 (30.2)	154 (19.0)	<0.001***
30–44	870 (45.24)	495 (44.6)	375 (46.2)	
45–59	563 (29.28)	280 (25.2)	283 (34.9)	
Nationality				
Kuwaiti	1,712 (89.0)	988 (88.9)	724 (89.2)	0.87
Non-Kuwaiti	211 (11.0)	123 (11.1)	88 (10.8)	
Governorate				
Capital	331 (17.21)	197 (17.7)	134 (16.5)	<0.001***
Ahmadi	470 (24.44)	309 (27.8)	161 (19.8)	
Farwaniya	239 (12.43)	116 (10.4)	123 (15.2)	
Hawali	451 (23.45)	249 (22)	202 (24.9)	
Jahra	92 (4.78)	47 (4.23)	45 (5.5)	
Mubarak Alkabeer	340 (17.68)	193 (17.4)	147 (18.1)	
Type of housing				
Rented house/flat	554 (28.81)	299 (26.9)	255 (31.4)	0.035*
Flat at parent’s house	528 (27.46)	326 (29.3)	202 (24.9)	
Own house	841 (43.73)	486 (43.7)	355 (43.7)	
Size of household				
One person	27 (1.4)	10 (0.9)	17 (2.1)	<0.001***
Two people	93 (4.84)	65 (5.85)	28 (3.5)	
Three to five people	601 (31.25)	378 (34)	223 (27.5)	
Six people or more	1,202 (62.51)	658 (59.2)	544 (67.0)	
Education recode				
High school or less	206 (10.75)	139 (12.6)	67 (8.3)	0.002**
College Diploma or more	1,711 (89.25)	966 (87.4)	745 (91.8)	
Work status				
Employed	1,338 (69.58)	736 (66.3)	602 (74.1)	<0.001***
Student	293 (15.24)	196 (18)	97 (12.0)	
Retired/Unemployed	292 (15.18)	179 (16)	113 (13.9)	
Infected with COVID-19				
No	1,608 (84.19)	1,062 (96.7)	546 (67.2)	<0.001***
Yes	302 (15.81)	36 (3.28)	266 (32.8)	

*p-value<0.05, **p-value<0.01, ***p-value<0.001; *p*-values for categorical variables obtained by Chi-squared test.

**Figure 1:**
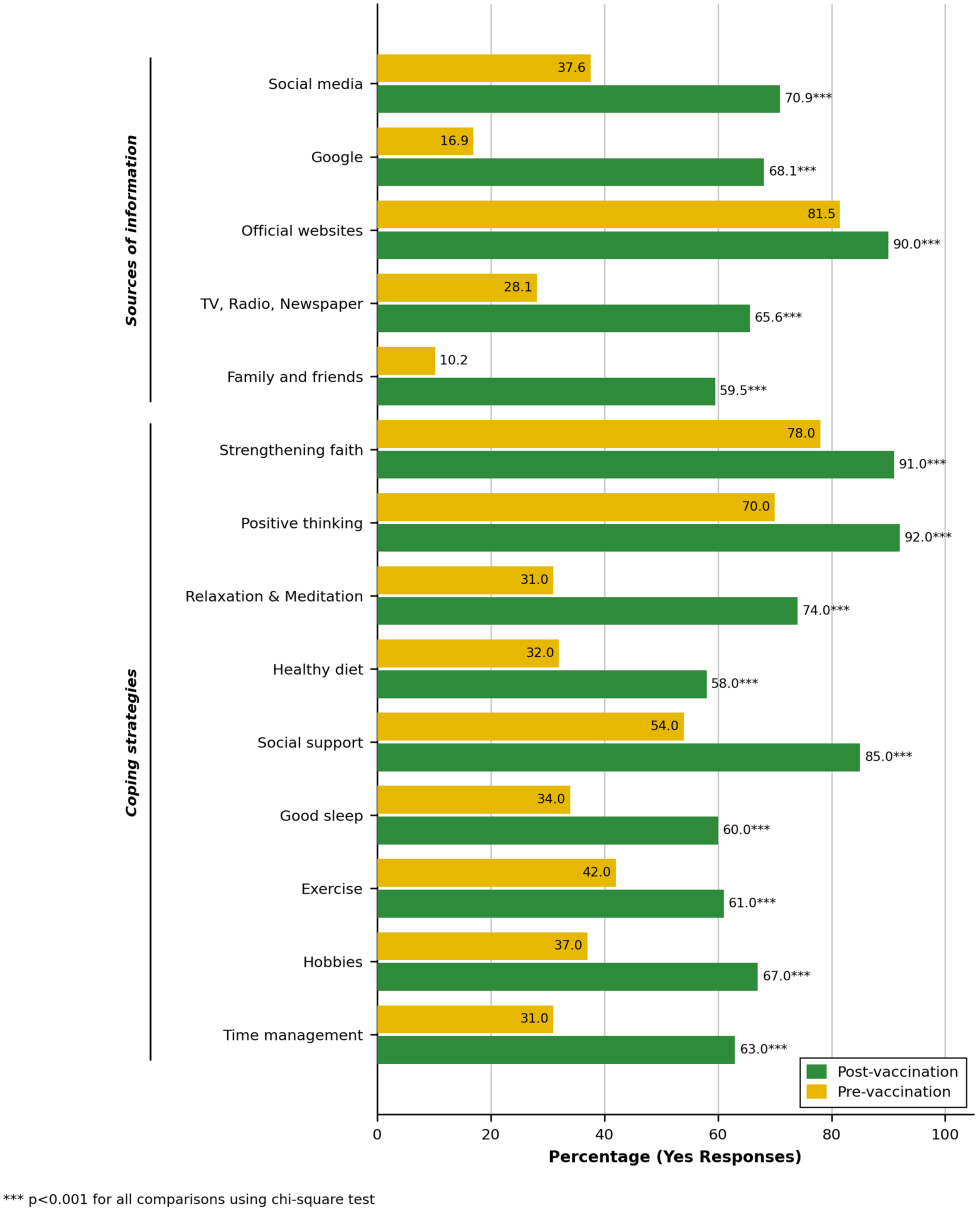
Differences in sources of information and coping strategies during the pre- and post-vaccination phases of the COVID-19 pandemic in Kuwait (June 2020–August 2021).

As shown in Figure 1, the proportion of participants who reported using official websites as a main source of COVID-19 information increased significantly from the pre- to post-vaccination period (82 to 90%, *p* < 0.001), remaining the most frequently cited source in both periods. Substantial increases were also observed in the use of Google (17 to 68%), social media (38 to 71%), and family and friends (10 to 60%) as information sources (*p* < 0.001 for all).

A significantly greater proportion of participants reported engaging in coping strategies during the post-vaccination period compared to the pre-vaccination period. The most notable increases were in the practice of relaxation and meditation (31 to 74%), time management (31 to 63%), and seeking social support (54 to 85%) (*p* < 0.001 for all). Improvements were also seen in other strategies, including maintaining a healthy diet, ensuring good sleep, and engaging in hobbies or physical activity.

**Figure 2:**
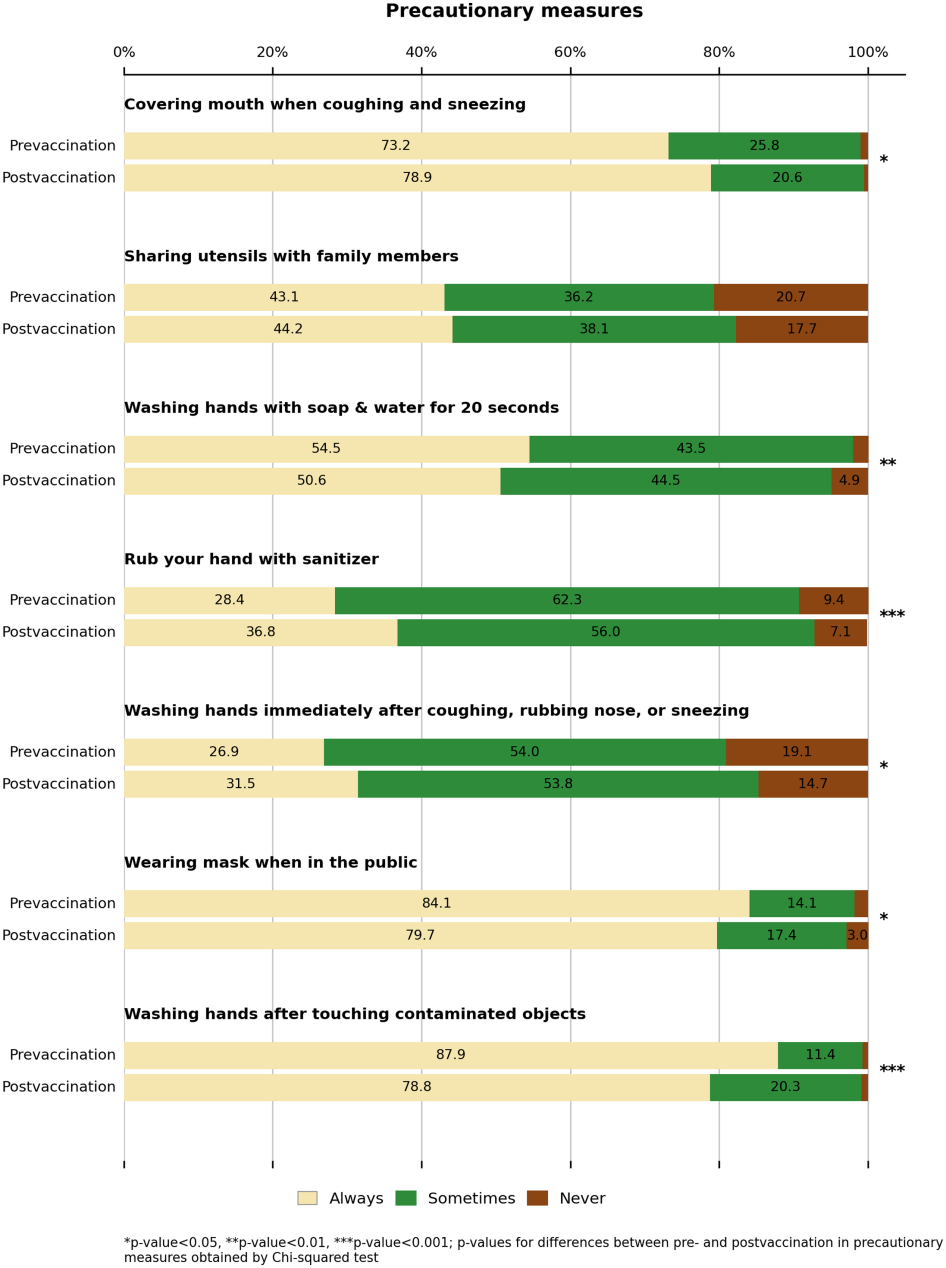
Changes in self-reported adherence to precautionary measures pre- and post COVID-19 vaccine availability in Kuwait (June 2020–August 2021).

Figure 2 illustrates significant changes in adherence to COVID-19 precautionary behaviors between the pre- and post-vaccination periods, with mixed patterns observed. Although certain practices, such as covering the mouth when coughing (79% vs. 73%) and using hand sanitizer (37% vs. 28%), increased significantly (*p* < 0.05), others declined. Most notably, fewer adults reported always washing their hands after touching contaminated objects (79% vs. 88%) or wearing masks in public (80% vs. 84%) in the post-vaccination period (*p* < 0.001 and *p* < 0.05, respectively). Overall, while some precautionary behaviors showed slight declines, most remained consistently practiced, indicating continued public adherence to COVID-19 hygiene measures even after vaccine availability.

### Concerns and mental health trends during the pandemic

**Figure 3:**
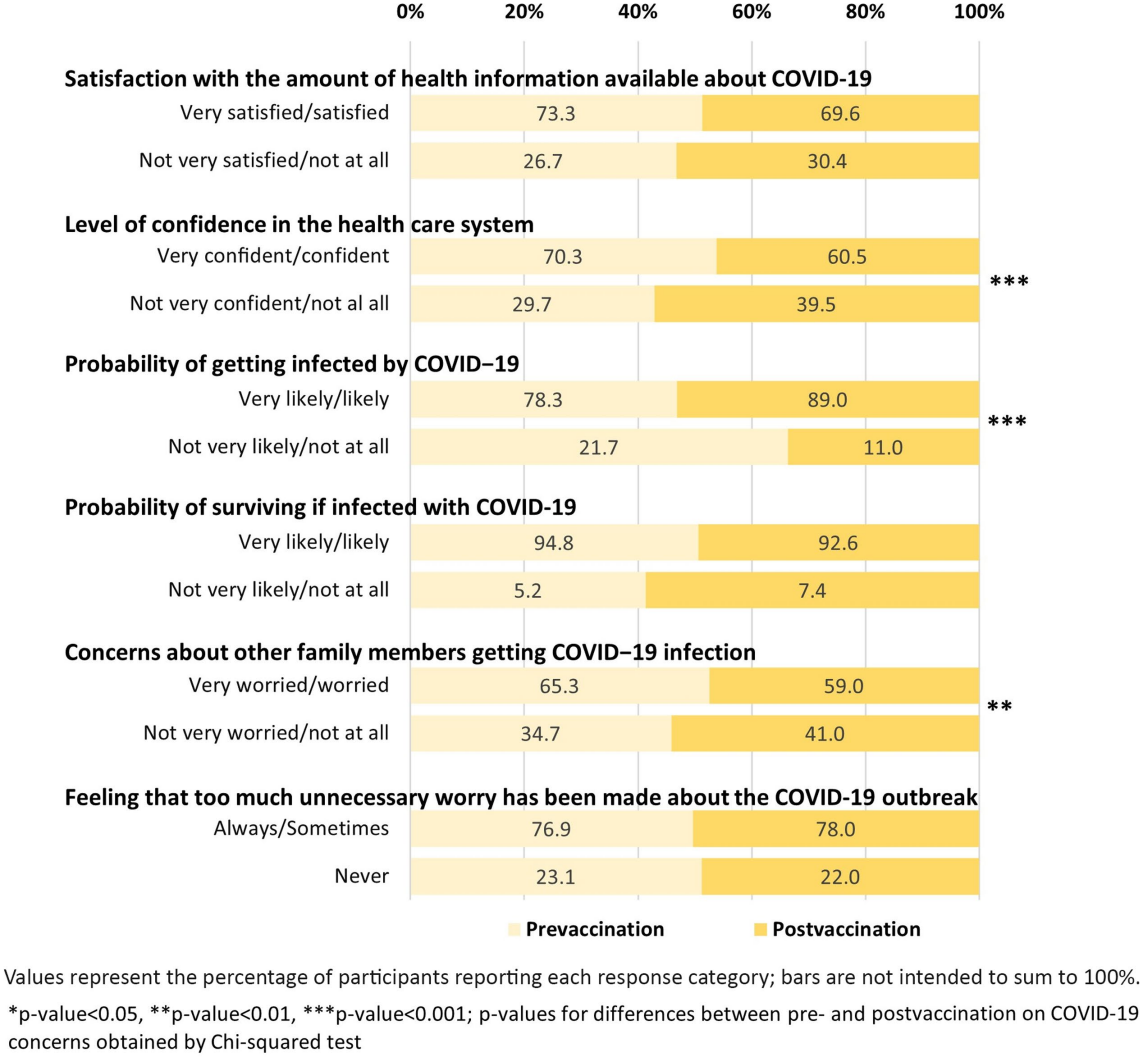
Changes in public concerns about COVID-19 during the pre- and post-vaccination periods in Kuwait (June 2020–August 2021).

Figure 3 illustrates that confidence in the healthcare system declined post-vaccination, with fewer participants reporting being “very confident” or “confident” (61% vs. 70%, *p* < 0.001). Perceived likelihood of infection increased significantly, with 89% of participants in the post-vaccination period reporting it was “likely” or “very likely” they would be infected, compared to 78% pre-vaccination (*p* < 0.001).

Concern for family members contracting the virus also declined (59% vs. 65%, *p* < 0.01), while perceptions of survival if infected and satisfaction with health information remained relatively stable. There were no significant differences in perceptions of whether unnecessary worry had been made about the outbreak (Figure 3).

**Figure 4:**
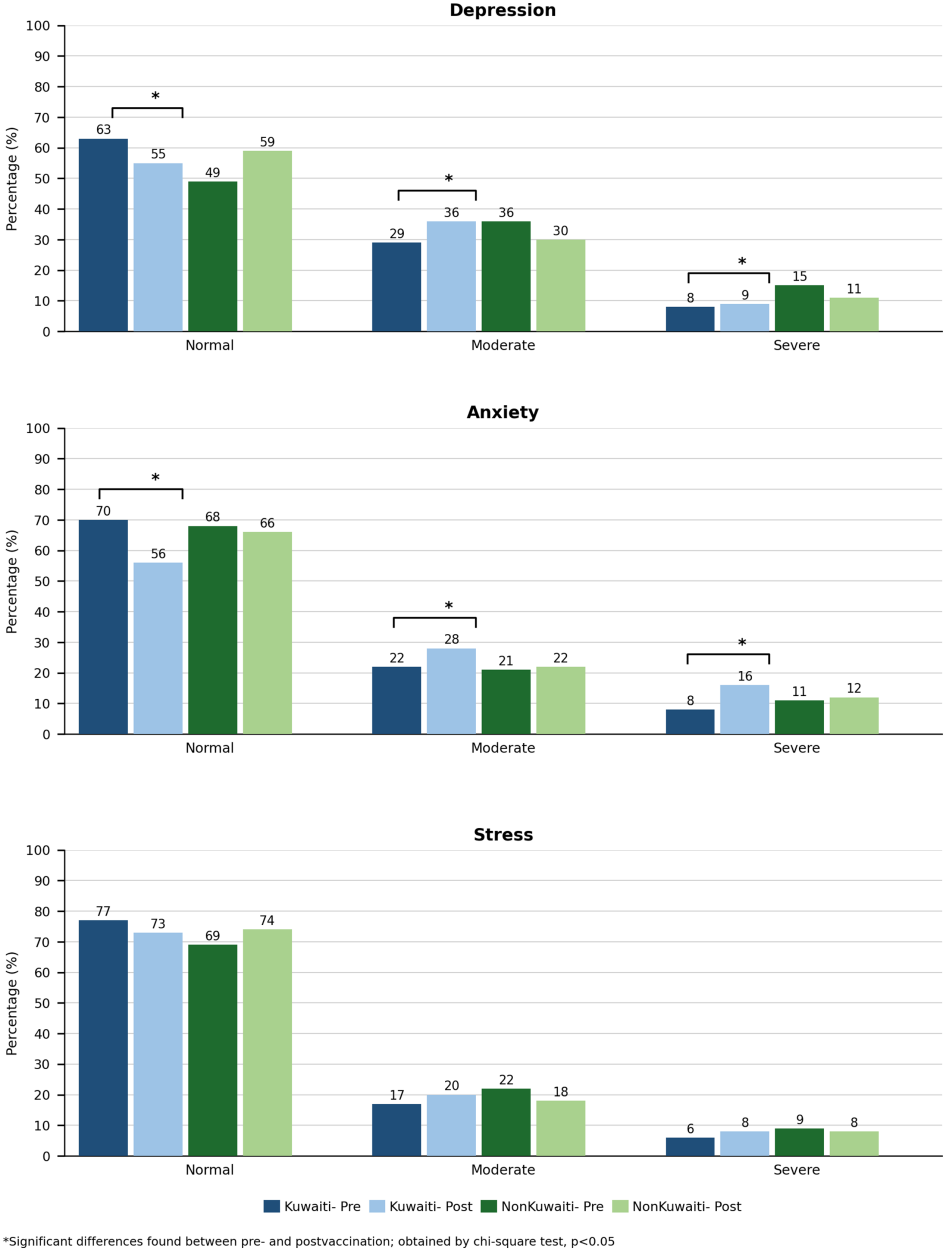
Mental health status by nationality and vaccination period during the COVID-19 pandemic in Kuwait (2020–2021, *n* = 1,923).

Significant shifts in mental health status were observed between the pre- and post-vaccination periods, particularly among Kuwaiti participants (Figure 4). The proportion of Kuwaitis with the normal range scores declined from 63 to 55% (*p* < 0.05), while moderate depression increased from 29 to 36% (*p* < 0.05). Severe depression also rose significantly among non-Kuwaitis (8 to 15%, *p* < 0.05).

For anxiety (Figure 4), normal levels among Kuwaitis declined from 70 to 56% (*p* < 0.05), accompanied by a significant rise in both moderate (22 to 28%) and severe anxiety (8 to 16%, *p* < 0.05). No significant differences were observed in unadjusted stress levels between pre- and post-vaccination periods for either group. These findings suggest that mental health concerns, particularly anxiety and depression, remained substantial throughout the pandemic.

Females were significantly more likely than males to report moderate or severe symptoms across all three mental health domains (*p* < 0.001 for all) (Table 2). Younger adults (aged 18–29) had the highest prevalence of moderate and severe anxiety and stress, with 16.3% reporting severe anxiety and 9.8% reporting severe stress (*p* < 0.001).

**Table 2:** Association between participant characteristics and depression, anxiety, and stress levels during the COVID-19 pandemic in Kuwait (pre- and post-vaccination, *n* = 1,923).

Variables	Total	Depression			Stress			Anxiety		
	*N* (%)	No %	Moderate %	Severe %	No %	Moderate %	Severe %	No %	Moderate %	Severe %
Total	1,923 (100)	59.1	31.8	9.2	64.2	24.3	11.4	74.8	18.5	6.7
Data collection										
Prevaccination	1,111 (57.8)	61.7	29.4	8.9	69.2	22.1	8.7***	76.3	17.7	5.9***
Postvaccination	812 (42.2)	55.5	35.0	9.5	57.4	27.5	15.2	72.8	19.6	7.6
Gender										
Male	876 (45.6)	68.2	26.5	5.4***	71.0	22.6	6.4***	80.8	15.2	4.0***
Female	1,047 (54.5)	51.5	36.2	12.3	58.6	25.8	15.7	69.8	21.3	8.9
Age (years)										
18–29	490 (25.5)	49.0	34.5	**	55.7	28.0	16.3***	65.9	24.3	9.8***
30–44	870 (45.2)	57.9	34.0	8.1	62.0	24.8	13.2	72.9	19.8	7.4
45–59	563 (29.3)	69.6	25.9	4.4	75.1	20.4	4.4	85.6	11.6	2.8
Nationality										
Kuwaiti	1,712 (89.0)	59.8	31.5	8.6*	63.9	24.7	11.4	75.3	18.3	6.4
Non-Kuwaiti	211 (11.0)	53.1	33.7	13.3	66.8	21.3	11.9	71.1	20.4	8.5
Governorate										
Capital	331 (17.2)	57.1	32.9	10.0**	65.0	21.5	13.6	71.0	21.8	7.3
Ahmadi	470 (24.4)	63.8	30.6	5.5	66.2	23.8	10.0	80.4	14.3	5.3
Farwaniya	239 (12.4)	54.0	33.9	12.1	58.2	26.4	15.5	73.2	20.1	6.7
Hawali	451 (23.5)	54.1	33.9	12.0	65.2	24.2	10.6	72.3	20.4	7.3
Jahra	92 (4.8)	58.7	27.2	14.1	57.6	29.4	13.0	70.7	17.4	12.0
Mubarak Alkabeer	340 (17.7)	64.7	29.1	6.2	65.6	25.3	9.1	76.5	17.9	5.6
Type of housing										
Rented house/flat	554 (28.8)	56.3	32.9	10.8	60.1	25.1	14.8*	70.8	20.0	9.2**
Flat at parent’shouse	528 (27.5)	60.6	31.6	7.8	63.5	25.6	11.0	73.3	20.1	6.6
Own house	841 (43.7)	59.9	31.2	8.9	67.4	23.1	9.5	78.5	16.5	5.0
Size of household										
One person	27 (1.4)	66.7	11.1	22.2	63.0	18.5	18.5	70.4	11.1	18.5
Two people	93 (4.8)	50.5	39.8	9.7	68.8	21.5	9.7	76.3	16.1	7.5
Three to five people	601 (31.3)	60.7	31.0	8.3	65.6	24.1	10.3	76.0	18.0	6.0
Six people or more	1,202 (62.5)	58.7	32.0	9.2	63.2	24.8	12.0	74.2	19.1	6.7
Education recode										
High school or less	206 (10.8)	55.8	31.6	12.6	63.6	20.4	16.0	72.8	17.0	10.2
College Diploma ormore	1,711 (89.3)	59.5	31.9	8.7	64.3	24.8	10.9	75.1	18.6	6.3
Work status										
Employed	1,338 (69.6)	60.0	31.5	8.5***	63.0	25.9	11.1***	74.8	18.2	7.0**
Student	293 (15.2)	47.8	35.8	16.4	57.3	24.2	18.4	67.2	24.2	8.5
Retired/Unemployed	292 (15.2)	66.1	29.1	4.8	76.7	17.5	5.8	82.5	14.0	3.4
Infected with COVID-19										
No	1,608 (84.2)	60.8	30.4	8.8**	67.6	22.3	10.1***	76.1	17.5	6.4*
Yes	302 (15.8)	51.0	37.8	11.3	47.7	35.4	16.9	69.2	22.9	8.0

*p-value < 0.05, **p-value < 0.01, ***p-value < 0.001; *p*-values for categorical variables obtained by Chi-squared test.

Non-Kuwaitis reported significantly higher rates of severe depression (13.7%) compared to Kuwaitis (8.5%, *p* < 0.05), while students exhibited the highest prevalence of moderate to severe anxiety (35.2%) and stress (30.1%) compared to other work status categories (*p* < 0.001) (Table 2). Individuals with a history of COVID-19 infection also reported significantly more severe anxiety and stress symptoms compared to those who had not been infected (*p* < 0.001 and *p* = 0.04, respectively).

### Multivariable predictors of mental health outcomes

#### Depression

In the adjusted multivariable logistic regression model (Table 3), participants in the post-vaccination period had significantly higher odds of moderate-to-severe depression compared to those surveyed pre-vaccination (OR = 1.74, 95% CI: 1.26–2.39, *p* = 0.001). Depression was more likely among females (OR = 1.65, 95% CI: 1.25–2.20, *p* = 0.001) and non-Kuwaitis (OR = 1.58, 95% CI: 1.02–2.44, *p* = 0.039), and less likely among participants aged 45–59 versus 18–29 years (OR = 0.45, 95% CI: 0.31–0.66, *p* < 0.001).

**Table 3:** Multivariable logistic regression analysis of factors associated with moderate-to-severe depression during the pre- and post-COVID-19 vaccination periods in Kuwait.

Characteristic	Depression (*N* = 1,161)	
	OR (95% CI)	*p*-value
Data collection period		
Prevaccination	[Reference]	
Postvaccination	**1.74** (1.26–2.39)	**0.001**
Gender		
Male	[Reference]	
Female	**1.65** (1.25–2.20)	**0.001**
Age (years)		
18–29	[Reference]	
30–44	**0.62** (0.45–0.86)	**0.005**
45–59	**0.45** (0.31–0.66)	**<0.001**
Nationality		
Kuwaiti	[Reference]	
Non-Kuwaiti	**1.58** (1.02–2.44)	**0.039**
Social media		
No	[Reference]	
Yes	**1.56** (1.17–2.07)	**0.002**
Level of confidence in the health care system		
Very confident/confident	[Reference]	
Not very confident/not confident at all	**1.83** (1.38–2.42)	**<0.001**
Sharing utensils with family members		
Always	[Reference]	
Sometimes	1.05 (0.78–1.43)	0.730
Never	**0.67** (0.45–0.99)	**0.043**
Washing hands immediately after coughing, rubbing nose, or sneezing		
Always	[Reference]	
Sometimes	1.18 (0.85–1.64)	0.312
Never	**1.94** (1.27–2.98)	**0.002**
Feeling that too much unnecessary worry has been made about COVID-19		
Always	[Reference]	
Sometimes	**0.44** (0.31–0.63)	**<0.001**
Never	**0.22** (0.14–0.34)	**<0.001**
Strengthening the faith factor		
No	[Reference]	
Yes	**0.64** (0.45–0.91)	**0.014**
Positive thinking and optimism		
No	[Reference]	
Yes	**0.40** (0.28–0.57)	**<0.001**
Social support		
No	[Reference]	
Yes	**0.72** (0.53–0.99)	**0.042**
Practicing good time management		
No	[Reference]	
Yes	**0.60** (0.45–0.81)	**0.001**

Use of social media as a main information source was associated with higher odds of depression (OR = 1.56, 95% CI: 1.17–2.07, *p* = 0.002), as was low confidence in the healthcare system (OR = 1.83, 95% CI: 1.38–2.42, *p* < 0.001). In contrast, protective coping strategies included positive thinking (OR = 0.64, 95% CI: 0.45–0.91, *p* = 0.014), social support (OR = 0.60, 95% CI: 0.52–0.99, *p* = 0.042), good time management (OR = 0.60, 95% CI: 0.45–0.81, *p* = 0.001), and regular physical activity (OR = 0.72, 95% CI: 0.56–0.92, *p* = 0.008).

#### Anxiety

Participants surveyed post-vaccination had significantly higher odds of moderate-to-severe anxiety (Table 4) compared to those surveyed pre-vaccination (OR = 2.14, 95% CI: 1.61–2.85, *p* < 0.001). Females were more likely than males to report anxiety (OR = 1.65, 95% CI: 1.29–2.11, *p* < 0.001), and participants aged 45–59 had significantly lower odds than those aged 18–29 (OR = 0.48, 95% CI: 0.34–0.68, *p* < 0.001).

**Table 4:** Multivariable logistic regression analysis of factors associated with moderate-to-severe anxiety during the pre- and post-COVID-19 vaccination periods in Kuwait.

Characteristic	Anxiety (*N* = 1,557)	
	OR (95% CI)	*p*-value
Data collection period		
Prevaccination	[Reference]	
Postvaccination	**2.14** (1.61–2.85)	**<0.001**
Gender		
Male	[Reference]	
Female	**1.65** (1.29–2.11)	**<0.001**
Age (years)		
18–29	[Reference]	
30–44	0.83 (0.61–1.11)	0.203
45–59	**0.48** (0.34–0.68)	**<0.001**
Nationality		
Kuwaiti	[Reference]	
Non-Kuwaiti	0.98 (0.67–1.44)	0.924
Infected with COVID-19		
No	[Reference]	
Yes	**1.70** (1.23–2.35)	**0.001**
Satisfaction with the amount of health information available about COVID-19		
Very satisfied/ satisfied	[Reference]	
Not very satisfied/ Not satisfied at all	**1.51** (1.14 -1.98)	**0.004**
Level of confidence in the health care system		
Very confident/confident	[Reference]	
Not very confident/not confident at all	**1.35** (1.03–1.76)	**0.027**
Concerns about other family members getting COVID−19 infection		
Very worried/ worried	[Reference]	
Not very worried/ not worried at all	**0.72** (0.56–0.94)	**0.014**
Covering mouth when coughing and sneezing		
Always	[Reference]	
Sometimes	**1.42** (1.07–1.88)	**0.014**
Never	**2.03** (0.57–7.20)	**0.273**
Sharing utensils with family members		
Always	[Reference]	
Sometimes	**1.33** (1.02–1.74)	**0.033**
Never	0.75 (0.53–1.05)	0.096
Washing hands with soup and water for 20 s		
Always	[Reference]	
Sometimes	1.52 (1.10–2.09)	0.011
Never	**4.88** (1.11–21.42)	**0.036**
Feeling that too much unnecessary worry has been made about COVID-19		
Always	[Reference]	
Sometimes	**0.33** (0.25–0.45)	**<0.001**
Never	**0.15** (0.10–0.23)	**<0.001**
Positive thinking and optimism		
No	[Reference]	
Yes	**0.69** (0.51–0.94)	**0.017**
Exercising or playing sports		
No	[Reference]	
Yes	**0.72** (0.56–0.92)	**0.008**
Practicing good time management		
No	[Reference]	
Yes	**0.70** (0.54–0.92)	**0.010**

Participants with lower satisfaction regarding available health information (OR = 1.51, 95% CI: 1.14–1.98, *p* = 0.004) and lower confidence in the healthcare system (OR = 1.35, 95% CI: 1.03–1.76, *p* = 0.027) had higher odds of anxiety. Those who were less concerned about family members getting infected had lower odds of anxiety (OR = 0.72, 95% CI: 0.56–0.94, *p* = 0.014). Among precautionary behaviors, inconsistent face-mask use (OR 1.42, 95% CI 1.07–1.88, *p* = 0.014) and washing hands with soap only sometimes (OR 1.52, 95% CI 1.10–2.09, *p* = 0.011) were associated with higher odds of anxiety. Coping strategies such as strengthening faith (OR = 0.42, 95% CI: 0.24–0.74, *p* = 0.003), positive thinking (OR = 0.69, 95% CI: 0.51–0.94, *p* = 0.017), and time management (OR = 0.70, 95% CI: 0.54–0.92, *p* = 0.010) were associated with lower odds of anxiety.

#### Stress

Participants during the post-vaccination period had higher odds of reporting moderate-to-severe stress (Table 5) compared to the pre-vaccination period (OR = 2.31, 95% CI: 1.73–3.07, *p* < 0.001), with females also more likely than males to experience stress (OR = 1.69, 95% CI: 1.30–2.21, *p* < 0.001). Older adults aged 45–59 had significantly lower odds than those aged 18–29 (OR = 0.39, 95% CI: 0.27–0.56, *p* < 0.001).

**Table 5:** Multivariable logistic regression analysis of factors associated with moderate-to-severe stress during the pre- and post-COVID-19 vaccination periods in Kuwait.

Characteristic	Stress (*N* = 1,790)	
	OR (95% CI)	*p*-value
Data collection period		
Prevaccination	[Reference]	
Postvaccination	**2.31** (1.73–3.07)	**<0.001**
Gender		
Male	[Reference]	
Female	**1.69** (1.30–2.19)	**<0.001**
Age (years)		
18–29	[Reference]	
30–44	0.75 (0.56–1.01)	0.061
45–59	**0.39** (0.27–0.56)	**<0.001**
Governorate		
Capital	[Reference]	
Ahamdi	**0.53** (0.36–0.79)	**0.002**
Farwaniya	**0.63** (0.41–0.97)	**0.038**
Hawalli	0.89 (0.61–1.29)	0.527
Jahra	0.70 (0.38–1.29)	0.250
Mubarak	**0.65** (0.44–0.97)	**0.036**
Type of housing		
Rented apartment	[Reference]	
Rented house	0.80 (0.47–1.36)	0.416
Apartment at parent’s house	0.72 (0.50–1.03)	0.072
Own house	**0.65** (0.46–0.93)	**0.018**
Level of confidence in the health care system		
Very confident/confident	[Reference]	
Not very confident/not confident at all	**1.57** (1.23–2.01)	**<0.001**
Concerns about other family members getting COVID−19 infection		
Very worried/ worried	[Reference]	
Not very worried/ not worried at all	**0.53** (0.40–0.69)	**<0.001**
Covering mouth when coughing and sneezing		
Always	[Reference]	
Sometimes	**1.42** (1.08–1.87)	**0.012**
Never	1.76 (0.50–6.26)	0.382
Feeling that too much unnecessary worry has been made about COVID-19		
Always	[Reference]	
Sometimes	**0.55** (0.41–0.74)	**<0.001**
Never	**0.30** (0.20–0.45)	**<0.001**
Positive thinking and optimism		
No	[Reference]	
Yes	**0.54** (0.40–0.73)	**<0.001**
Social support		
No	[Reference]	
Yes	**0.70** (0.53–0.92)	**0.011**
Sleep		
No	[Reference]	
Yes	**0.70** (0.53–0.91)	**0.008**
Hobbies		
No	[Reference]	
Yes	**0.72** (0.55–0.94)	**0.015**
Practicing good time management		
No	[Reference]	
Yes	**0.73** (0.55–0.96)	**0.025**

Compared with the Capital governorate, residence in Ahmadi (OR 0.63, *p* = 0.002), Farwaniya (OR 0.55, *p* < 0.001), or Mubarak Al-Kabeer (OR 0.65, *p* = 0.036) was associated with lower odds of stress. Lower confidence in the healthcare system was associated with higher odds of stress (OR = 1.57, 95% CI: 1.23–2.01, *p* < 0.001), while lower concern about family members’ infection was associated with lower stress levels (OR = 0.53, 95% CI: 0.40–0.69, *p* < 0.001).

Protective coping strategies for stress included strengthening faith (OR = 0.30, 95% CI: 0.20–0.45, *p* < 0.001), social support (OR = 0.54, 95% CI: 0.40–0.73, *p* < 0.001), good sleep (OR = 0.70, 95% CI: 0.52–0.93, *p* = 0.011), optimism (OR = 0.70, 95% CI: 0.53–0.91, *p* = 0.008), engaging in hobbies (OR = 0.72, 95% CI: 0.55–0.94, *p* = 0.015), and time management (OR = 0.73, 95% CI:0.55–0.96, *p* = 0.025).

## Discussion

This study examined mental health outcomes among adults in Kuwait across two phases of the COVID-19 pandemic: the pre-vaccine and post-vaccine periods. Using the 21-item Depression, Anxiety, and Stress Scale (DASS-21), a validated and cross-culturally reliable instrument (14, 17), we compared psychological wellbeing between two independent survey samples. The findings provide insight into population-level differences in mental health across distinct pandemic phases and inform future outbreak preparedness planning.

Across both survey periods, a substantial proportion of participants reported symptoms of depression (40.9%), anxiety (36.0%), and stress (25.0%). After adjustment for sociodemographic and behavioral factors, participants surveyed during the post-vaccine phase had higher odds of moderate-to-severe anxiety compared with those surveyed during the pre-vaccine phase. These findings reflect differences between two independent population samples rather than within-person change. Observed differences may be influenced by compositional variation between survey waves, as well as broader contextual factors present during each phase of the pandemic, including evolving public health policies, economic pressures, and ongoing uncertainty. Similar patterns of sustained psychological distress across successive pandemic phases have been reported in longitudinal studies from Italy and Japan (18, 19), highlighting the importance of maintaining mental health support beyond the acute stage of public health emergencies.

Female sex and younger age (18 to 29 years) were strongly associated with higher odds of depression, anxiety, and stress. These findings align with evidence from the Gulf region and international studies showing that women experienced disproportionately higher psychological distress during the pandemic (5, 16, 20). In Kuwait, female sex was similarly identified as a risk factor for depression and anxiety during the early pandemic period (21). Biological and psychosocial factors may contribute to women’s greater vulnerability to mood and anxiety disorders (22, 23).

Younger adults may have been particularly affected due to disrupted routines, prolonged social isolation, and greater exposure to online misinformation (24, 25). Although some studies have reported heightened psychological risk among older adults, our findings suggest that extended family structures common in Kuwait may have provided social support that buffered older individuals against severe mental health effects. Together, these results highlight the importance of age- and gender-sensitive mental health strategies in future public health crises.

Non-Kuwaiti participants had significantly higher odds of depression compared to Kuwaitis, even after adjustment for potential confounders. This finding is consistent with evidence indicating that migrant workers may face employment instability and barriers to accessing health services, vulnerabilities that were highlighted during the COVID-19 pandemic (26). Given Kuwait’s demographic structure and large expatriate population, addressing mental health inequities among non-Kuwaitis is essential for an inclusive and effective public health response.

Use of informal information sources increased substantially during the post-vaccine phase, including Google, social media, and family or friends, although official websites remained the most trusted source overall. This pattern of multiple simultaneous sources mirrors early COVID-19 findings where government websites were also dominant alongside diverse usage (27), while hesitant adopters reported similar reliance on personal relationships (28). Higher trust in social media as an information source has been associated with increased psychological burden during the pandemic (29).

Positive coping strategies were reported more frequently during the post-vaccine phase, particularly relaxation, meditation, and time management. Multivariable analyses showed that faith-based practices, optimism, social support, time management, and physical activity were associated with lower odds of depression, anxiety, and stress. These findings are consistent with prior research demonstrating the protective role of resilience and social support (30), as well as the mediating role of adaptive coping in reducing mental health problems (31). Conversely, these results support evidence that maladaptive or negative coping styles are significant predictors of increased psychological distress (32). Given their cultural relevance and accessibility, these strategies may be especially valuable in contexts where formal mental health services are limited. Integrating coping guidance into public health messaging could strengthen future crisis responses.

In Kuwait, these findings suggest that preparedness strategies could formally incorporate culturally relevant coping resources. Partnerships with religious institutions and community organizations may help disseminate structured coping guidance during crises. Embedding brief stress-management messages within official Ministry of Health communication channels, particularly on widely used social media platforms, could further support population resilience, especially among women, younger adults, and non-citizens identified as higher-risk groups.

Differences in preventive behaviors were observed between survey phases. While covering the mouth when coughing and using hand sanitizer were reported more frequently during the post-vaccine phase, consistent mask use and hand washing after touching contaminated objects were less frequently reported. These patterns may reflect shifts in risk perception, as individuals can demonstrate risk tolerance even when a threat is recognized as serious (33). Reduced perception of personal threat in the post-vaccine era may also have contributed to risk compensation, whereby protective behaviors decline as the environment is perceived to be safer (34). These findings are further consistent with pandemic fatigue, defined as gradual demotivation to adhere to recommended protective behaviors over time (35). Together, they underscore the need for sustained behavioral messaging regardless of vaccine status.

Public confidence in the healthcare system was lower during the post-vaccine phase compared with the pre-vaccine phase. Although confidence remained relatively high, this decline may reflect evolving expectations and frustrations as the pandemic progressed. Prior research indicates that confidence in healthcare systems and trust in government are associated with greater adherence to recommended preventive behaviors during the COVID-19 pandemic (36, 37). In our study, lower confidence was associated with higher levels of depression, anxiety, and stress, underscoring trust as an important correlate of population mental health. Maintaining transparent, responsive, and equitable healthcare communication should therefore remain a priority in future public health emergencies.

Several limitations should be considered. The repeated cross-sectional design precludes causal inference and does not permit assessment of within-person changes over time. Recruitment through convenience-based online sampling may have introduced selection bias, potentially underrepresenting individuals with limited digital access, lower socioeconomic status, or greater social marginalization. In addition, significant differences in sociodemographic characteristics between the two survey waves indicate compositional variation, which may partially account for observed differences in mental health outcomes despite multivariable adjustment. Although models adjusted for measured covariates, residual confounding cannot be excluded, and findings may not be fully generalizable to vulnerable populations underrepresented in online recruitment. Nevertheless, the large sample size, inclusion of two distinct pandemic phases, and use of a validated instrument strengthen the credibility and relevance of the findings.

## Conclusion

This study demonstrates substantial levels of psychological distress across pre- and post-vaccine phases of the COVID-19 pandemic. By identifying key risk groups, including women, younger adults, and non-citizens, and highlighting the protective role of culturally embedded coping strategies such as faith and social support, the findings offer actionable insights for future pandemic preparedness. To strengthen health system resilience, future crisis responses in Kuwait should integrate mental health into public health planning through sustained, inclusive, and context-sensitive interventions, including structured coping guidance and targeted outreach to higher-risk groups identified in this study.

## Data Availability

Data may be made available by the corresponding author upon reasonable request, subject to approval by the relevant institutional ethics authorities. Requests to access the datasets should be directed to Office of the Assistant Undersecretary for Planning and Quality Affairs, hkhamis@moh.gov.kw.
